# Determinants of delays in travelling to an emergency obstetric care facility in Herat, Afghanistan: an analysis of cross-sectional survey data and spatial modelling

**DOI:** 10.1186/s12884-015-0435-1

**Published:** 2015-02-05

**Authors:** Atsumi Hirose, Matthias Borchert, Jonathan Cox, Ahmad Shah Alkozai, Veronique Filippi

**Affiliations:** PhD programme, Faculty of Epidemiology and Population Health, London School of Hygiene & Tropical Medicine, London, UK; Faculty of Epidemiology and Population Health, London School of Hygiene & Tropical Medicine, London, UK; Institute of Tropical Medicine and International Health, Charité – Universitätsmedizin, Berlin, Germany; Faculty of Infectious and Tropical Diseases, London School of Hygiene & Tropical Medicine, London, UK; World Vision International, Herat, Afghanistan

**Keywords:** Afghanistan, Delays, Emergency obstetric care, Referrals, Maternal health, Near-miss, Geographic information systems, Social network, Transportation

## Abstract

**Background:**

Women’s delays in reaching emergency obstetric care (EmOC) facilities contribute to high maternal and perinatal mortality and morbidity in low-income countries, yet few studies have quantified travel times to EmOC and examined delays systematically. We defined a delay as the difference between a woman’s travel time to EmOC and the optimal travel time under the best case scenario. The objectives were to model travel times to EmOC and identify factors explaining delays. i.e., the difference between empirical and modelled travel times.

**Methods:**

A cost-distance approach in a raster-based geographic information system (GIS) was used for modelling travel times. Empirical data were obtained during a cross-sectional survey among women admitted in a life-threatening condition to the maternity ward of Herat Regional Hospital in Afghanistan from 2007 to 2008. Multivariable linear regression was used to identify the determinants of the log of delay.

**Results:**

Amongst 402 women, 82 (20%) had no delay. The median modelled travel time, reported travel time, and delay were 1.0 hour [Q1-Q3: 0.6, 2.2], 3.6 hours [Q1-Q3: 1.0, 12.0], and 2.0 hours [Q1-Q3: 0.1, 9.2], respectively. The adjusted ratio (AR) of a delay of the “one-referral” group to the “self-referral” group was 4.9 [95% confidence interval (CI): 3.8-6.3]. Difficulties obtaining transportation explained some delay [AR 2.1 compared to “no difficulty”; 95% CI: 1.5-3.1]. A husband’s very large social network (> = 5 people) doubled a delay [95% CI: 1.1-3.7] compared to a moderate (3-4 people) network. Women with severe infections had a delay 2.6 times longer than those with postpartum haemorrhage (PPH) [95% CI: 1.4-4.9].

**Conclusions:**

Delays were mostly explained by the number of health facilities visited. A husband’s large social network contributed to a delay. A complication with dramatic symptoms (e.g. PPH) shortened a delay while complications with less-alarming symptoms (e.g. severe infection) prolonged it. In-depth investigations are needed to clarify whether time is spent appropriately at lower-level facilities. Community members need to be sensitised to the signs and symptoms of obstetric complications and the urgency associated with them. Health-enhancing behaviours such as birth plans should be promoted in communities.

## Background

Women’s delay in reaching emergency obstetric care (EmOC) facilities contributes to a high burden of maternal and perinatal mortality and morbidity in low-income countries. Basic EmOC (BEmOC) services dispense life-saving functions such as the administration of antibiotics, oxytocic drugs, and anticonvulsants, as well as manual removal procedures, while comprehensive EmOC (CEmOC) services additionally include blood transfusion and surgery. The period from the onset of signs and symptoms of complications to the receipt of EmOC is usually divided into three phases or “three delays” [1]: The first delay refers to the interval between the onset of obstetric complication and the decision to seek care; the second delay is the interval between the decision and the arrival in a health facility; and the third delay is between the arrival and the provision of adequate care. Each interval has a distinctive set of factors determining its duration. Factors prolonging the second interval include travel distance [2,3], sparsely distributed EmOC health facilities (particularly in rural areas) [4], ineffective referrals [5,6], a lack of transportation means [3,6,7], the cost of transportation [8], and drivers’ unwillingness to transport women in labour. While many studies provide these factors as reasons for the second delay, few empirical studies have attempted to quantify travel distances and times for women needing EmOC [4,9].

In recent years, the increased availability of geo-referenced data has made it easier to calculate distances between patients’ residences and the locations of care and hence provide a direct measure of geographical accessibility [10-13]. Since many women reach an EmOC facility in life-threatening condition after considerable delay [14,15], detailed analyses of distances and travel times are warranted for this particular group of women. We postulate that the use of geo-referenced data may create new knowledge of care-seeking behaviours which contribute to travel delay in complicated pregnancies and childbirth.

Afghanistan has one of the highest maternal mortality ratios in the world, much of which is explained by women’s difficult physical access and socio-cultural barriers to care [16]. The country is very mountainous with a rudimentary road network and limited means of transportation. Due to threats by anti-government elements and insecurity, vehicle drivers may be unwilling to transport pregnant women, particularly in rural areas. Furthermore, the Afghan society is based on a strong patriarchy, where women are viewed as “receptacles of honour” [17]. To protect family honour, a woman’s mobility is controlled by her male relatives [17], limiting her access to health care. Women are often brought to EmOC facilities in moribund conditions after considerable access delay [18].

We undertook the current study to understand the travel delays of women who were in a life-threatening condition at admission to a large maternity hospital in Afghanistan. The study operationalised in statistical terms the second phase of delay presented in Thaddeus and Maine’s seminal work [1]. Specific objectives were to (1) estimate the distance and travel time to a CEmOC facility using geo-referenced data in a geographic information system (GIS); (2) assess factors associated with the travel times modelled in GIS and reported by these women; and (3) explore factors explaining the differences between the modelled and reported travel times.

## Methods

### Study setting

**Figure 1:**
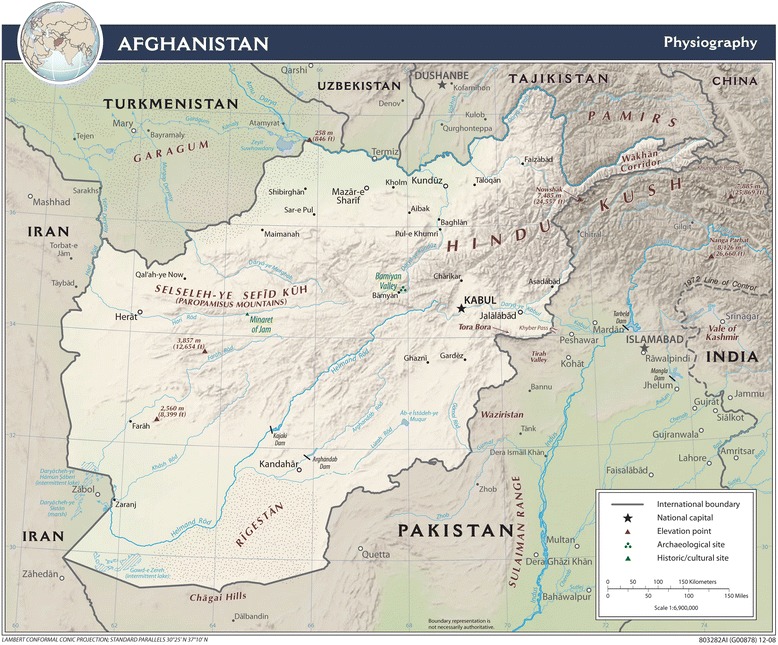
**“Afghanistan physiography” 2009.** *Source:* U.S. Central Intelligence Agency [19].

The study was set in the western region of Afghanistan, which includes Herat, Badghis, Ghor, and Farah provinces. Despite decades of conflict, the city of Herat, the provincial capital and location of our study hospital, is well developed in terms of basic infrastructure compared with other parts of the country. Paved roads connect the provincial capital to Turkmenistan via the border town of Turgundi in the north (2 hours by vehicle) and to the Islamic Republic of Iran via Islam Qala in the west (1.5-2 hours by vehicle). East of Herat and at the end of the Hindu Kush (a mountain range stretching between central Afghanistan and northern Pakistan) is Ghor province; its capital, Chaghcharan, is at about 2500 m above sea level, a much higher altitude than Herat (Figure 1). In both Ghor and Badghis (northeast of Herat) provinces, transportation links are particularly poor due to rugged terrain and the lack of paved roads, and existing roads are often blocked by snowfall in winter. South of Herat is sparsely populated Farah province, with half of its land being mountainous. Herat Regional Hospital was the only CEmOC facility in western Afghanistan.

### Data collection

Empirical travel times were collected during a cross-sectional survey of women admitted to the maternity ward of Herat Regional Hospital in a life-threatening condition and of their male relatives between February 2007 and January 2008. Details of the survey are presented elsewhere [18]. In short, we recruited prospectively all the women meeting disease-specific criteria of “near-miss” morbidity at admission during the study period. The disease-specific criteria of “near-miss” were adapted from other studies conducted in resource-limited settings [20-22]. Face-to-face interviews were conducted mostly before discharge, except for four interviews conducted at home with female relatives who cared for four women who died in hospital. A wide range of topics was covered during the interview, amongst which the residence of the woman’s birth family and the utilization of health care during pregnancy were considered in this particular analysis. From the male relative (usually the husband), we obtained information on departure time from home and arrival time at the study hospital and, if relevant, at lower health facilities; access to and utilization of transportation means; family composition; household assets; his occupation and education status; his participation in community activities; the size of his social network; the village of residence; and a nearby notable village (for the ease of village identification).

### Estimation of Euclidian distance

To obtain the geographical coordinates of each woman’s village, we used a settlement database provided by the Afghanistan Information Management Services (AIMS, available at http://www.aims.org.af/). The woman’s reported village of residence was manually identified in the database, and its coordinates extracted. Herat Hospital’s geographical coordinates were obtained with a handheld global positioning system (GPS) receiver (eTrex, Garmin [KS, USA]). Point locations for villages and Herat Hospital were imported into a GIS (ArcGIS version 10; CA, USA), and then into a raster-based GIS (IDRISI Andes, Clark Lab, MA, USA), to compute the Euclidian (straight-line) distance from a woman’s village of residence to Herat Hospital.

### Modelling of travel time in a GIS

Travel times between individual residences or compounds and Herat Hospital were predicted with a cost-surface modelling approach in IDRISI. This method involves assigning “friction” values to represent the land surface types that either impede or facilitate travel. We considered travel speeds by the most suitable local transportation means under optimal conditions (best-case scenario). Vehicle travel speeds along the transportation network were estimated based on observations and discussions with local drivers (80 km/h on primary roads; 55 km/h on secondary roads; and 40 km/h on tracks). A walking speed of 1.75 km/h was assumed for areas traversable by foot only [13]. Water bodies were deemed to be essentially impassable (very close to 0 km/h).

AIMS spatial data on the road network, land cover, and drainage were used to derive a composite friction surface for the study area with a spatial resolution (i.e., pixel size) of 100 m. A cost-surface grid was then derived, indicating the minimum accumulative travel time between each compound and the hospital. Initial results from this exercise were compared with travel times to 12 major villages in the study area as reported by experienced NGO drivers (“reference time”, shown in Table 1). We preferred to rely on drivers’ reports (instead of having them travel just to measure the time), because of insecurity in the area and the need to restrict travel to the essential. The deviations of the modelled times from the reference times were greatest in mountain areas. Hence, we decided to incorporate an additional barrier, slope angle, so that steeply inclined surfaces would be assigned higher friction values than flat surfaces. After iterations with different friction values, estimated travel times to 12 major villages reached reasonable values (Table 1). The travel time was then extracted for the residence of each woman in our sample.

**Table 1:** **Model validation results: reference and modelled travel times by major destination**

**Village/area of residence**	**District**	**Province**	**Features of the roads between the village and Herat Hospital**	**Reference time (minutes)**	**Initial model results (minutes)**	**Final model results (minutes)**
Guzara district centre	Guzara	Herat	Paved roads	15	21.5	21.5
Qala-i-Naw district centre	Qala-i-Naw	Badghis	All types of roads passing through mountains	240-300	188.9	244.7
Karukh district centre	Karukh	Herat	Paved roads	30-40	38.8	38.8
Turghundi	Kushk	Herat	Paved roads but not very smooth	120	93.7	93.7
Islam Qala	Kohsan	Herat	Paved roads only	90-100	97.1	97.1
Shahrak district centre	Shahrak	Ghor	In the mountains	540-600	304.7	415.1
Dara-Takht	Chishiti Sherif	Herat	Tracks	360	244.9	250.9
Shindand district centre	Shindand	Herat	Paved roads	100-120	128.3	130.3
Chishiti Sherif district centre	Chishiti Sherif	Herat	Tracks	270	208.4	208.4
Obe district centre	Obe	Herat	Tracks but relatively flat	150-180	162.4	162.5
Pashtun Zarghun district centre	Pashtun Zarghun	Herat	Tracks but relatively flat	90	117.2	117.2
Chartaz	Jawand	Badghis	All types of roads passing through mountains	720	383.4	728.6

### The definition of a delay and the variables in the conceptual framework

For the remaining part of this paper, we defined a delay as the difference between the travel time computed from interview results and the expected travel time derived from the GIS model [1]. We postulated that consultation times at lower-level health facilities would inevitably prolong the travel time to the final CEmOC facility. Additionally, we hypothesised that social, relational, environmental, and obstetric factors would affect access to and the use of transportation between home and health facilities and cause delays. Variables were selected for each of these themes (Table 2). The first set of variables was intended to capture the social class hierarchy in a rural society. Those with greater social, economic, or political power in the rural community often have a greater command over the transportation business and services [23,24]. Not only do scarce financial resources hamper the rural poor from using transportation, the poor may also be charged higher rates for sub-optimal services, because the low demand in rural villages does not promote fair competition [25]. Three proxy variables for social class were included: (1) Asset-based household socio-economic status (created by adding weights equal to the inverse of the proportion of households owning selected household items and creating quartiles); (2) the husband’s occupation group; and (3) the husband’s education (whether he has ever attended formal school). Literature suggests that a woman’s education is positively associated with her utilisation of health care services and health-promoting behaviours. Nonetheless, we postulated that the husband’s education would have a stronger influence than the woman’s, because of the cultural context in which male relatives chaperone women, the urgency of the situations, and the fact that some of the women became very ill quickly after signs and symptoms were recognised. Hence, the benefits of female education—such as an educated woman’s new values and attitudes (e.g., enhanced self-confidence, self-worth) that are favourable to her use of health care and her increased discussion with her husband [26]—may not have a strong influence on the travel to a health facility in the context of this study.

**Table 2:** **Variables considered in the analysis**

**1. Referral**	**2. Social class hierarchy**	**3. Relational factors**	**4. Environmental factors**	**5. Obstetric factors**	**6. Transportation factors**
No. health facilities before admission to CEmOC	Asset-based socio-economic status	Husband’s participation in community activities in last 12 months	Season	Complication type	Transferred in an ambulance
	Husband’s occupation	No. people the husband can rely on to borrow a small amount of money from	Urban/rural	Antenatal care utilization	Community usually has a vehicle
	Husband’s education	No. people the husband can rely on in long-term emergency		Parity	Reported difficulty obtaining a vehicle
		Family type (nuclear vs. extended family)			
		Woman’s birth family lives nearby			

The second set of variables was intended to measure the size and strength of the husband’s social network. When travel entails a long distance, families with greater social networks may more easily overcome financial and logistical barriers to transportation. Three variables identified from the group and network section of a social capital questionnaire [27] were considered: (1) The husband’s participation in community activities in the last 12 months; (2) the number of people the husband reports to be able to rely on in case of long-term emergency (such as a job loss or harvest failure); and (3) the number of people the husband reports to be able to borrow a small amount of money from. Two additional variables, household type (extended or nuclear family) and distance to the woman’s birth family, were included. In Afghanistan, as in other countries in South Asia, “the patriarchal extended family is the central social unit” [28], and the society is based on the institutionalized relationships of mutual dependency among the patriarchal kin. Members of the patriarchal kinship network help each other during hard times, emotionally or materially. The woman’s birth family can be another important source of support around the time of childbirth. We hypothesised that couples living with their extended family or close to the woman’s birth family have better access to material resources.

Third, two variables were selected to capture environmental factors. Concerning the season of admission, we hypothesised that travel in winter and summer may take longer due to road damage or obstruction by rain or snow during the winter, and to difficulties travelling in the midday sun during the summer. We included urban or rural residence as an independent variable because of two possible scenarios: Urban residents may experience a delay due to city congestion, while residents in remote areas may experience a delay because of vehicle maintenance on the way.

Fourth, we considered maternal and obstetric factors and selected three variables: Complication type, parity, and the number of antenatal care (ANC) visits. A woman’s complications with typically dramatic symptoms (i.e., eclampsia and postpartum haemorrhage) and her past experience of childbirth or lack of it may reinforce care-takers’ perception of the urgency of the situation, and the woman may be transported to a health facility as rapidly as possible.

Finally, we included three variables that indicate access to and utilization of vehicles, as they directly affect travel times to the hospital: Transfer in an ambulance; the community normally has a vehicle; and reported difficulty obtaining transportation.

### Data analysis

Survey data and travel distance and time data created in IDRISI were imported into Stata (version 9; Stata Corp, TX, USA) for analysis. Euclidian distances, GIS-modelled travel times, and self-reported travel times were described by key determinants. Because these data were skewed, the Kruskal-Wallis test was used, and medians plus inter-quartile ranges presented. Delay, the main outcome of the study, was expressed as the difference between the reported travel time and the GIS-modelled travel time. The delay data were log-transformed to achieve a normal distribution after a minimal value was added to the data to adjust for non-positive delays. We used multivariable regression methods with forward selection to identify determinants, and likelihood ratio tests (p < 0.05) to decide on the inclusion of a variable in the model. Final results were reverted to normal scale and presented as ratios to the baseline group.

### Ethics clearance

Ethics approval was obtained from the ethics committees of the Ministry of Public Health in Afghanistan and the London School of Hygiene & Tropical Medicine. Individual informed consent was obtained from all women and husbands who agreed to participate in the study. Either a thumb-print or a signature was obtained.

## Results

Of the 472 women and their husbands recruited for the study, 410 couples were included in the current analysis. Sixty-two couples were excluded because the exact location of their village was unknown for various reasons: The male relative did not participate in the interview (33 women); nomads *(kuchi)* did not know where they were staying when complications were recognised (4 women); or the village name reported during the interview did not match any village in the AIMS database (25 women). Eight were further excluded from analysis because their reported travel times were at least 1 hour shorter than the modelled travel time, which we deemed implausible. Finally, there were 23 observations with missing exposure data due to no response. This resulted in the total of 387 observations included in the final model. Evidence suggests that included and excluded women were different: More excluded women were from the poorest quartile than included women (72.2% vs. 26.1%). All except some Herat City and nearby Injil district residents had access to a vehicle at one point during the care-seeking process.

### Euclidian distance

**Figure 2:**
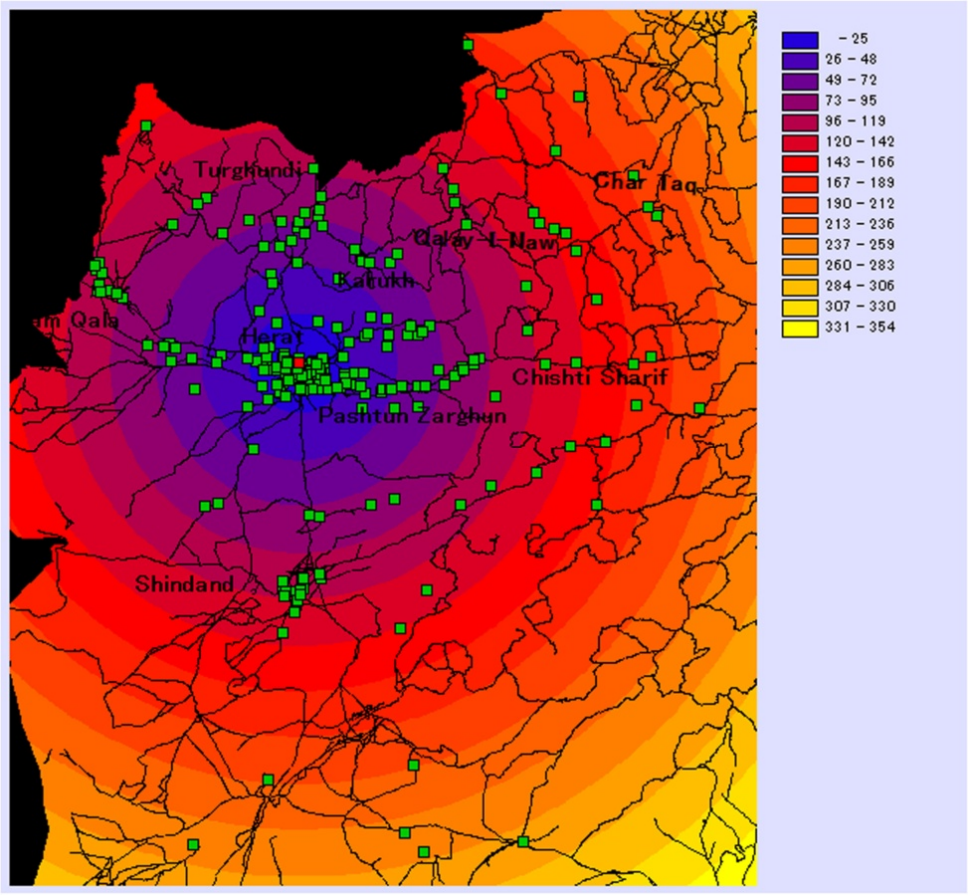
**Map showing Euclidian (straight line) distances in km the patients’ residences to Herat Hospital.** *Note:* The red square on the map indicates the location of Herat Hospital. Green squares indicate the villages of the women in our sample.

The entire sample lived within a radius of 268 km from the hospital, with a median Euclidian distance of 21 km (Figure 2). Median distances differed significantly by complication type: While women with severe pre-eclampsia and haemorrhage in early pregnancy tended to come from relatively close neighbourhoods (median = 7.8 km and 10.4 km, respectively), women with impending rupture of the uterus, ruptured uterus, or severe infection travelled on average from much farther communities (median = 52.3 km, 65.7 km, and 85.0 km, respectively). Those transferred in an ambulance came from more distant communities than those procuring their own transportation means (median = 76.9 km vs. 15.1 km; p < 0.0001). Those reporting difficulty obtaining transportation travelled significantly farther that those reporting no difficulty (median = 80.7 km vs. 7.8 km). The poorest women travelled from significantly farther away than the least poor women (median = 80.7 km vs. 0.4 km). The indicators of social network or season did not show a strong association with distance (Table 3).

**Table 3:** **Distance, modelled travel time, reported travel time, and delay, by risk factors**

***Risk factors***	**N**	**Straight-line distance (km) [IQ range]**	p-value*	**N**	**Modelled travel time (hours) [IQ range]**	p-value*	**N**	**Reported travel time (hours) [IQ range]**	p-value*	**N**	**Delay (hours) [IQ range]**	p-value*
***The number of health facilities visited before CEmOC***												
2 or more	69	79.2 [21.4, 116.5]		69	2.1 [1.0, 4.1]		67	29.5 [8.2, 88.0]		67	24.9 [7.0, 84.3]	
1	173	48.6 [10.8, 98.9]		174	1.6 [0.6, 2.7]		172	5.3 [3.1, 13.7]		172	3.8 [1.4, 11.5]	
None (came straight to CEmOC)	159	7.8 [0.4, 18.0]	<0.0001	159	0.6 [0.6, 1.0]	<0.0001	158	0.8 [0.5, 1.5]	<0.0001	158	0 [-0.2, 0.4]	<0.0001
***Complication type***												
Haemorrhage in early pregnancy	60	10.4 [0.4, 37.9]		60	0.7 [0.6, 2.0]		58	4.5 [1.0, 29.5]		58	2.5 [0.2, 28.9]	
Severe pre-eclampsia	51	7.8 [0,4, 33.9]		51	0.6 [0.6, 1.3]		50	3.0 [0.7, 26.5]		50	1.9 [0.1, 23.1]	
APH	55	16.3 [2.9, 89.0]		55	0.9 [0.6, 2.3]		54	3.4 [0.8, 8.0]		54	1.5 [0.3, 5.6]	
Eclampsia	93	27.1 [3.1, 84.1]		93	1.1 [0.6, 2.5]		93	3.5 [1.0, 9.0]		93	2.2 [0.1, 4.9]	
Impending rupture of the uterus	38	52.3 [14.1, 98.9]		38	1.6 [0.9, 2.7]		38	6.4 [1.5, 14.3]		38	4.6 [0.3, 11.1]	
Rupture of the uterus	30	65.7 [7.8, 106.3]		30	1.6 [0.6, 2.5]		29	5.0 [1.0, 10.0]		29	2.8 [0.6, 7.5]	
PPH	48	21.3 [10.1, 63.9]		48	1.0 [0.6, 1.9]		47	1.3 [0.5, 4.0]		47	0.2 [-0.1, 1.2]	
Sepsis	23	85.0 [9.7, 155.3]		24	2.6 [0.7, 7.8]		24	21.3 [2.8, 68.5]		24	12.4 [1.9, 54.9]	
Other	4	74.4 [23.8, 109.9]	<0.0001	4	3.1 [1.7, 8.4]	0.0041	3	16.3 [2.0, 27.0]	0.0003	4	9.1 [0.8, 19.1]	<0.0001
***Parity***												
6 or more	90	19.9 [5.9, 75.0]		90	1.0 [0.6, 2.3]		87	4.0 [1.0, 13.0]		87	2.2 [0.3, 10.7]	
3-5	96	14.2 [0.4, 71.4]		96	0.8 [0.6, 1.9]		94	2.7 [0.7, 9.5]		94	1.4 [0.1, 6.9]	
1-2	78	48.6 [9.9, 106.3]		78	1.6 [0.6, 2.7]		78	5.0 [1.2, 14.5]		78	2.9 [0.2, 9.4]	
Nullipara	109	11.7 [0.4, 71.7]	0.0078	109	0.8 [0.6, 2.0]	0.0608	108	3.0 [0.5, 10.5]	0.1786	108	1.6 [-0.1, 8.1]	0.3409
***Antenatal Care***												
No ANC	123	43.5 [7.1, 101.6]		123	1.6 [0.6, 3.3]		123	5.0 [1.5, 17.0]		123	3.0 [0.2, 13.8]	
1-3 visits	161	21.5 [4.1, 79.2]		161	1.0 [0.6, 1.9]		159	3.5 [1.0, 9.8]		159	2.2 [0.1, 6.8]	
4 or more visits	91	9.0 [0.4, 50.9]	0.0005	91	0.6 [0.6, 1.6]	0.0002	88	1.9 [0.5, 6.8]	0.0032	88	0.6 [-0.1, 5.4]	0.0390
***Reported difficulty in obtaining transportation***												
Very difficult	59	80.7 [37.2, 133.4]		59	3.1 [1.4, 6.6]		57	12.0 [4.0, 27.0]		57	6.4 [1.6, 20.8]	
Moderately difficult	124	34.9 [12.0, 93.3]		124	1.4 [0.6, 2.6]		122	4.4 [1.0, 10.0]		122	2.1 [0.4, 7.0]	
Not difficult	211	7.8 [0.4, 48.6]	<0.0001	211	0.6 [0.6, 1.6]	<0.0001	210	2.5 [0.5, 8.0]	<0.0001	210	1.2 [-0.1, 5.9]	<0.0001
***Community access to a vehicle***												
No	44	82.7 [30.4, 143.1]		44	3.3 [1.1, 7.1]		42	12.3 [4.5, 34.2]		42	6.6 [0.6, 33.2]	
Yes	354	16.5 [0.4, 75.0]	<0.0001	354	0.9 [0.6, 2.0]	<0.0001	350	3.0 [1.0, 10.0]	<0.0001	350	1.6 [0.1, 7.0]	0.0005
***Ambulance use***												
Yes	61	76.9 [41.2, 106.3]		61	2.1 [1.4, 3.1]		60	6.8 [4.0, 13.4]		60	4.6 [2.3, 10.9]	
No	337	15.1 [0.4, 71.7]	<0.0001	337	0.9 [0.6, 1.9]	<0.0001	333	2.8 [0.7, 11.0]	<0.0001	333	1.4 [0.0, 8.4]	0.0002
***Seasons***												
Summer	88	25.9 [0.4, 93.3]		88	1.0 [0.6, 2.6]		86	5.7 [1.0, 15.0]		86	4.0 [0.3, 13.4]	
Fall	102	17.2 [2.3, 75.8]		102	1.0 [0.6, 2.2]		102	3.0 [0.5, 7.0]		102	1.3 [0.1, 5.4]	
Winter	104	22.3 [0.4, 83.0]		105	1.0 [0.6, 1.9]		104	3.8 [1.0, 11.6]		104	1.9 [0, 8.4]	
Spring	108	21.5 [5.9, 78.9]	0.8922	108	1.1 [0.6, 2.3]	0.9072	105	3.5 [1.0, 9.5]	0.1564	105	1.8 [0.2, 6.5]	0.1403
***Household socio-economic status***												
Poorest	105	80.7 [48.3, 117.8]		105	2.5 [1.6, 4.4]		103	8.0 [4.0, 17.5]		103	4.9 [1.6, 14.6]	
2nd poorest	85	43.0 [16.3, 84.1]		85	1.7 [0.8, 2.6]		84	4.0 [1.3, 11.3]		84	1.8 [0.3, 8.8]	
3rd poorest	105	13.4 [3.4, 39.3]		105	0.7 [0.6, 1.5]		103	2.0 [0.5, 9.0]		103	1.2 [-0.1, 7.0]	
Least poor	107	0.4 [0.4, 7.8]	<0.0001	107	0.6 [0.6, 0.7]	<0.0001	106	1.0 [0.5, 5.0]	<0.0001	106	0.4 [-0.1, 3.9]	<0.0001
***Occupation group***												
Farming (including Kuchi/cattle herding)	136	65.0 [24.3, 113.3]		136	1.9 [1.0, 3.3]		135	6.0 [2.5, 18.5]		135	3.7 [0.6, 16.8]	
Business/shopowners/drivers	67	1.4 [0.4, 11.3]		67	0.6 [0.6, 0.9]		66	2.3 [0.5, 6.0]		66	0.8 [0.1, 5.4]	
Teachers, engineers, government officials	26	3.1 [0.4, 16.5]		26	0.6 [0.6, 1.0]		25	1.0 [0.5, 6.5]		25	0.4 [-0.1, 5.9]	
Labourers	152	16.4 [0.4, 63.7]		152	0.8 [0.6, 1.8]		149	2.0 [0.5, 8.0]		149	1.2 [-0.1, 5.4]	
Other	21	59.7 [4.1, 98.9]	<0.0001	21	1.8 [0.6, 2.1]	<0.0001	21	3.3 [1.2, 15.0]	<0.0001	21	1.4 [0.3, 6.4]	0.0007
***Husband’s education***												
No	246	33.8 [6.9, 87.0]		246	1.3 [0.6, 2.7]		243	4.5 [1.0, 13.0]		242	2.6 [0.2, 9.6]	
Yes	154	11.0 [0.4, 68.5]	0.0019	154	0.8 [0.6, 1.8]	0.0041	151	2.5 [0.7, 10.0]	0.0547	151	0.9 [0, 7.2]	0.1019
***Participation in community activities in the last 12 months***												
No	263	24.2 [3.4, 87.0]		263	1.1[ 0.6, 2.3]		260	4.5 [1.0, 14.8]		260	2.9 [0.2, 12.5]	
Yes	130	15.0 [3.1, 69.1]	0.2084	130	0.9 [0.6, 2.1]	0.1918	127	2.5 [0.8, 6.5]	0.0155	127	0.8 [0.1, 5.4]	0.0132
***Number of people the husband can borrow a small amount of money from***												
0	23	24.2 [9.7, 71.2]		22	1.1 [0.6, 2.9]		21	5.0 [1.0, 20.0]		12	4.3 [0.6, 16.2]	
1-2	137	33.7 [6.9, 84.1]		135	1.1 [0.6, 2.5]		134	4.5 [1.0, 13.0]		134	2.8 [0.2, 10.3]	
3-4	136	23.2 [5.5, 89.0]		137	1.2 [0.6, 2.1]		136	3.5 [1.0, 13.5]		136	2.0 [0.1, 10.1]	
5 or more	98	10.2 [0.4, 68.5]	0.0296	99	0.8 [0.6, 2.1]	0.0616	97	2.8 [0.8, 10.0]	0.3663	87	1.2 [0.1, 7.0]	0.5336
***Number of people the husband can rely on in long-term emergency***												
0	205	28.1 [5.7, 83.2]		205	1.1 [0.6, 2.7]		203	4.0 [1.0,14.0]		203	1.9 [0.2, 12.4]	
1-2	133	17.2 [0.4, 75.0]		133	0.9 [0.6, 1.9]		130	3.9 [1.0, 8.0]		130	2.0 [0, 5.7]	
3-4	22	15.6 [5.9, 94.5]		22	1.0 [0.6, 2.0]		22	2.0 [0.7, 5.5]		22	0.7 [0.1, 3.9]	
5 or more	36	9.8 [0.4, 98.7]	0.4958	36	0.9 [0.6, 2.0]	0.2261	35	8.0 [2.0, 34.5]	0.081	35	5.6 [0.6, 33.2]	0.0595
***Woman’s birth family lives nearby***												
No	83	27.1 [5.9, 82.0]		85	1.1 [0.6, 2.0]		84	4.6 [1.0, 14.8]		84	2.6 [0.1, 13.1]	
Yes	289	18.3 [0.4, 79.2]	0.3787	289	1.0 [0.6, 2.2]	0.5561	284	3.5 [1.0, 10.0]	0.2718	284	1.8 [0.2, 7.2]	0.5272
***Household type***												
Nuclear	220	16.5 [4.1, 76.3]		220	1.0 [0.6, 2.0]		214	3.1 [1.0, 11.0]		214	1.5 [0.1, 7.5]	
Extended	181	24.4 [2.3, 89.0]	0.3575	181	1.2 [0.6, 2.5]	0.2263	181	4.5 [1.0, 14.3]	0.165	181	2.8 [0.2, 10.7]	0.2774
***Residence***												
Rural	301	50.9 [16.0, 98.9]		302	1.6 [0.8, 2.7]		298	4.8 [1.5, 14.5]		298	2.9 [0.4, 12.4]	
Urban	101	0.4 [0.4, 0.4]	<0.0001	101	0.6 [0.6, 0.6]	<0.0001	99	0.5 [0.5, 4.5]	<0.0001	99	0.2 [-0.1, 3.9]	<0.0001

Note: APH = antepartum haemorrhage; CEmOC = comprehensive emergency obstetric care; PPH = postpartum haemorrhage; *P-values are from Kruskal-Wallis Test.

*P-values are from Kruskal-Wallis Test.

### Modelled travel time

**Figure 3:**
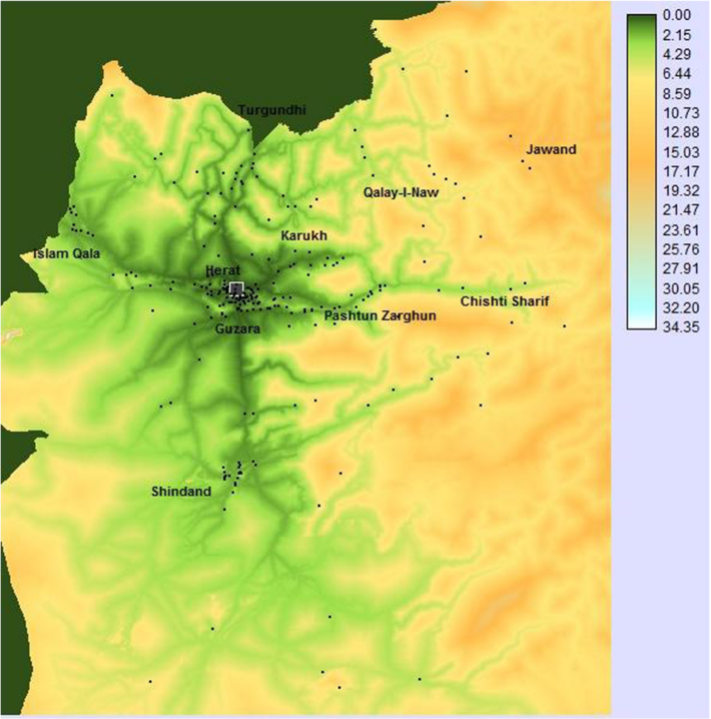
**Map showing estimated travel time in hours from patients’ residences to Herat Hospital.** *Note:* The grey square on the map indicates the location of Herat Hospital. Small dots indicate the villages of the women in our sample.

Under optimal conditions, median modelled travel time to the hospital was just over an hour [Q1-Q3: 0.6, 2.2], with a maximum of 16 hours (Figure 3). Differences by complication types and other determinants were observed, but in general, trends were similar to those observed for Euclidian distances (Table 3).

### Self-reported travel time

The median reported travel time to the hospital was 3.6 hours [Q1-Q3: 1.0, 12.0] (data not shown). Women with haemorrhage in early pregnancy and severe pre-eclampsia spent more time travelling to the hospital (4.5 hours and 3.0 hours, respectively) than women with PPH (1 hour), although the former group of women lived closer to the hospital. Half of the women with severe infection and with impending or actual rupture of the uterus spent over 20, 5, and 6 hours, respectively, travelling to the hospital. The median travel time for the poorest women was 8 hours vs. 1 hour for the least poor women. When the husband had participated in community activities, the woman’s median travel time was about 2 hours shorter than when he had not (Table 3).

### Bivariable and multivariable analyses of delay

The median delay (i.e., the difference between the reported and modelled travel time) was 2.0 hours [Q1-Q3: 0.1, 9.2]. For 82 women (20%), no positive delay was computed. The proportion of women without delay was greater among urban than rural residents (47% vs. 12%). Delays experienced by self-referring women were minimal, while women referred by a lower health facility had a delay of about 4 hours from their residence to the CEmOC. The delay was increased by over 20 hours when women were referred by two or more health facilities. On average, women with PPH had a shorter delay (0.2 hours) than women with other complication types (median delays for impending rupture of the uterus and severe infection were over 4 and 12 hours, respectively). Difficulty obtaining transportation or a community’s lack of a vehicle prolonged travel times by over 6 hours on average. The travel delay was shorter when the husband had participated in community activities in the last 12 months than when he had not (0.8 hours vs. 2.9 hours), but having 5 or more people to rely on in case of long-term emergency increased the delay by over 5 hours. Higher wealth quartile and increased ANC attendance were associated with a shorter delay (Table 3).

Multivariable analysis showed that a delay was prolonged by the number of health facilities visited (adjusted ratios (AR) of the “one-referral” group = 4.9; AR of the “2 or more referrals” group = 19.5, compared to the “self-referral” group). Reported difficulties obtaining transportation and the husband’s large (>5 people) social network doubled a delay. Certain complication types further prolonged a delay (Table 4).

**Table 4:** **Results of multivariable regression model to explain the magnitude of delay**

		**Adjusted ratio to the reference group [95% CI]**	**p-value**
***Number of facilities visited before CEmOC***			
	2 or more	19.5 [13.7, 27.8]	<0.0001
	1	4.9 [3.8, 6.4]	<0.0001
	None (CEmOC only)	Reference	
***Complication type***			
	Haemorrhage in early pregnancy	1.9 [1.2, 3.1]	0.006
	Severe pre-eclampsia	2.1 [1.3, 3.5]	0.002
	APH	1.6 [1.0, 2.6]	0.042
	Eclampsia	1.4 [0.9, 2.1]	0.111
	Impending rupture of the uterus	1.7 [1.0, 2.9]	0.032
	Rupture of the uterus	1.8 [1.1, 3.2]	0.032
	PPH	Reference	
	Severe infection	2.6 [1.4, 4.9]	0.002
	Other	0.7 [0.2, 2.2]	0.499
***Reported difficulties obtaining transportation***			
	Very difficult	2.1 [1.5, 3.0]	<0.0001
	Moderately difficult	1.0 [0.8, 1.3]	0.929
	Not difficult	Reference	
***Number of people the husband can rely on in long-term emergency***			
	0	1.4 [0.8, 2.3]	0.257
	1-2	1.2 [0.7, 2.1]	0.474
	3-4	Reference	
	5 or more	2.0 [1.1, 3.7]	0.033

Note: APH = antepartum haemorrhage; CEmOC = comprehensive emergency obstetric care; PPH = postpartum haemorrhage.

## Discussion

We showed that travel delay is a function of the number of health facilities visited, difficulty in obtaining transportation, complication type, and certain characteristics of a woman’s family members. These findings are in line with previous studies suggesting that the travelled distance alone does not necessarily indicate travel time [4].

The magnitude of a delay explained by the number of facilities visited was much greater than that of a delay explained by other factors (including transportation-related factors). For women living 1 km away from the hospital, visiting two or more health facilities is likely to be unnecessary. However, for residents of distant communities, it is unclear whether there was an unnecessary delay in visiting a health facility, as the time may have been spent receiving necessary first aid before being transferred to the CEmOC facility. The more complex a case, the more time that may be needed to provide care at a lower health facility. Detailed investigation into the kinds of care provided at lower health facilities (particularly information about the appropriateness of treatment provided for each complication type) would (a) help identify areas for improvement in the referral system, and (b) clarify to what extent seeking care in such facilities means a loss of time or an enhanced chance for a positive clinical outcome.

Women with severe infection experienced a much greater delay than women with PPH. This phenomenon may be explained by women and care-takers’ slow reactions to the less-alarming symptoms of infection compared with the often-dramatic PPH. Yet it is also possible that lower health facilities may not have managed all severe infection appropriately, causing a referral delay. It also suggests that women with haemorrhage may not have reached the hospital in time; PPH may be fatal within a few hours, while the interval between onset to death can be several days for puerperal infection [29]. Women with severe infection received care in the study hospital after travelling for many hours from distant communities, while women with haemorrhage travelled from relatively close neighbourhoods with a relatively short travel time. Women with severe pre-eclampsia also came from communities close to the hospital. This may be because women with severe pre-eclampsia from distant communities progressed to eclampsia before reaching the study hospital. On average, women presenting with eclampsia had come further afield than women presenting with pre-eclampsia. We also pondered the possibility that women with severe pre-eclampsia from distant communities had been successfully treated at lower health facilities in districts. Another possibility is that some women with severe pre-eclampsia died, whether or not they progressed to eclampsia, as Afghanistan has a relatively weak health system and high maternal mortality.

The literature on social capital has established a positive relationship between social capital (i.e., social cohesion, solidarity, and norms of trust and reciprocal support) and health [30]: Social capital promotes diffusion of health information and the adaptation of health-enhancing behaviour. Our finding that a husband’s large social network prolonged a woman’s delay in travelling to the hospital during an obstetric emergency contradicts such literature to some extent, and the negative aspects of social capital may need to be discussed. Some authors suggest that social capital may lead to diminished individual freedom and the exclusion of outsiders. Individuals may be encouraged to take up harmful behaviours fostered within the social network if these conform to community and cultural norms. Ogwang et al. report both helpful and harmful support that rural Ugandan communities would provide during obstetric emergencies, including referring the woman to a health facility, mobilizing money, and using a stretcher to carry the woman, but also taking the woman to a traditional healer [31]. Our study participants with a very large social network may have received confusing or contradictory advice, or they may have visited many members of their social network for financial, logistical, or emotional support. Our survey was unable to discern what kind of information was exchanged between community members, how resources were mobilised, and what role the male family member and his social network played. More work is needed to understand how social capital can negatively influence access to emergency care. In the meantime, programme implementers may need to ensure that health-enhancing behaviours are accepted and promoted within communities when encouraging men’s involvement in safe motherhood.

### Limitations

There are several methodological factors that likely contributed to the inaccuracy of the GIS modelled travel times. During interviews with urban participants, we learned their home district within the city of Herat, but the AIM’s database listed only two of the 10 districts. Residents of districts close to the hospital were assigned the coordinates of the AIM district that was closest to the hospital, and those living in farther urban districts were assigned the other. The use of a pixel size of 100 meters may not have captured the geographical complexity of urban areas. It may have been necessary to consider traffic flows and congestion in modelling urban travel times [32]. AIMS acknowledged that it was in the process of correcting geographical data at the time of the study; many settlements had moved due to the war, tribal disputes, and drought, and residents affected by such calamities are likely to be poorer. In particular, the relatively large difference between the reference time and the modelled travel time for Dara Takht village may be due partly to the incorrect geographical data entered into the model for this village. Our use of the driving times reported by the experienced NGO drivers as the reference may have introduced non-directional bias. All of these factors likely contributed to a mostly non-directional bias in modelled travel times. Modelled travel times from the mountainous areas east of Herat province needed improvement, but the number of women coming from this part of the region was too small for this inaccuracy to affect overall results.

Our study had other limitations. We relied on the husbands’ reports of departure times to compute “reported travel times”, which is a source of non-differential misclassification bias. Rural women who did not have access to a vehicle may have passed away before reaching the hospital, which may have led to a survival bias, possibly explaining why the magnitude of delay explained by transportation factors was relatively small in our study. We were unable to consider delays due to the inadequacy of the road network and how much reduction in the travel times could have been achieved from improving it.

Finally, our results may not be generalizable to all women experiencing severe obstetric complications in Afghanistan. However, we believe that they give a valid picture of the extent and the determinants of travel delays experienced by women admitted with near-miss obstetric complications to Herat Hospital. We decided to focus on women with near-miss complications rather than less severe forms of obstetric complications because their situations are the closest to maternal deaths.

## Conclusion

Reducing the second phase of delay is difficult for most maternal and child health programmes because it requires addressing multiple sectors: Transportation, communications, and infrastructure. Using both modelled and reported travel times, our study provides insights into non-infrastructural factors explaining travel delays. Much of a travel delay was due to the time women spent visiting lower-level health facilities, while transportation factors were responsible for only a small part. Investigation into what care was provided at BEmOC facilities is needed to understand whether time was lost by attending them first, or whether the time at the BEmOC facility was used sensibly to improve health outcomes. More research is needed to understand the mechanism of both the positive and negative effects of social networks, and programme implementers need to ensure that community male members possess appropriate health knowledge when the men are being involved in safe motherhood programmes. Methodological improvements may be needed to better model travel times in both urban and mountainous rural areas. Finally, although our study aimed to identify behavioural factors explaining travel delays that could be modified by health programmes, we also stress that a significant amount of travel time could probably be saved in the first place if the road network was improved and transportation options were increased in both the Herat region and throughout Afghanistan.
